# Identification of an osteoclastogenic CD4+ T cell population producing IL-17 and TNFα

**DOI:** 10.1186/1479-5876-9-S2-P24

**Published:** 2011-11-23

**Authors:** Thomas Ciucci, Eléonore Birgy-Barelli, Jérôme Pene, Grazia Abou-Ezzi, Nadia Arab, Xavier Hébuterne, Hans Yssel, Claudine Blin-Wakkach, Abdelilah Wakkach

**Affiliations:** 1 INSERM U576, Nice, France; 2 INSERM U844, Montpellier, France; 3 Dept. of Gastroenterology, Hospital l'Archet, Nice, France

## Background

Crohn's disease is an inflammatory bowel disease (IBD) characterized by an augmentation of activated T cells and a severe osteopenia due to an increased activity of osteoclasts, the bone-resorbing cells. Osteoclasts result from differentiation of monocytes under the control of two cytokines, RANK-L and M-CSF, produced by bone-forming osteoblasts. Inflammatory cytokines produced by T cells have been shown to increase osteoclatogenesis, leading to osteopenia. The aim of this study is to identify CD4+ T cells implicated in osteolysis.

## Material and methods

We used a murine model of IBD associated with severe osteopenia, the IL-10-/- mouse to analyze the phenotype and function of bone marrow (BM) CD4+ T cells.

## Results

We showed that CD4+ T cells isolated from the BM of IL-10-/- mice with IBD induced in vitro the differentiation of osteoclasts. The analysis in BM of the Th subsets present among these CD4+ T cells revealed, in addition to Th1, a population producing both TNF-α and IL-17, cytokines known to increase osteoclastogenesis. Our results showed that sorted CD4+ IL-17+ TNFα+ T cells increased the production of RANK-L by osteoblasts and induce the differentiation of osteoclasts in vitro. Furthermore, this population led to the recruitment of monocytes (pre-osteoclasts) in the BM in vivo participating thereby to the increased osteoclastogenesis. Altogether, the induction of RANK-L and monocyte recruitment by CD4+ IL-17+ TNFα+ T cells may represent a possible mechanism of osteolysis in vivo. Lastly, we found the CD4+ IL-17+ TNFα+ population in the blood of patients with Crohn's disease, but not in controls and experiments are in progress to confirm the osteoclastogenic role of this population in human.

## Conclusion

Altogether, our results showed for the first time that CD4+ IL-17+ TNFα+ cells represent an osteoclatogenic T cell subset present in vivo, and potentially responsible for osteopenia in mice and Crohn’s patients.

